# A simple analytical model for a fast 3D assessment of peripheral photon dose during coplanar isocentric photon radiotherapy

**DOI:** 10.3389/fonc.2022.872752

**Published:** 2022-10-06

**Authors:** Beatriz Sánchez-Nieto, Ignacio N. López-Martínez, José Luis Rodríguez-Mongua, Ignacio Espinoza

**Affiliations:** ^1^ Instituto de Física, Pontificia Universidad Católica de Chile, Santiago, Chile; ^2^ Departamento de Radiofísica, Fundación Arturo López Pérez, Santiago, Chile

**Keywords:** radiotherapy, photon peripheral dose, photon out-of-field dose, secondary cancer, stochastic radiation risk, Monte Carlo, analytical model, periphocal

## Abstract

Considering that cancer survival rates have been growing and that nearly two-thirds of those survivors were exposed to clinical radiation during its treatment, the study of long-term radiation effects, especially secondary cancer induction, has become increasingly important. To correctly assess this risk, knowing the dose to out-of-field organs is essential. As it has been reported, commercial treatment planning systems do not accurately calculate the dose far away from the border of the field; analytical dose estimation models may help this purpose. In this work, the development and validation of a new three-dimensional (3D) analytical model to assess the photon peripheral dose during radiotherapy is presented. It needs only two treatment-specific input parameter values, plus information about the linac-specific leakage, when available. It is easy to use and generates 3D whole-body dose distributions and, particularly, the dose to out-of-field organs (as dose–volume histograms) outside the 5% isodose for any isocentric treatment using coplanar beams [including intensity modulated radiotherapy and volumetric modulated arc therapy (VMAT)]. The model was configured with the corresponding Monte Carlo simulation of the peripheral absorbed dose for a 6 MV abdomen treatment on the International Comission on Radiological Protection (ICRP) 110 computational phantom. It was then validated with experimental measurements using thermoluminescent dosimeters in the male ATOM anthropomorphic phantom irradiated with a VMAT treatment for prostate cancer. Additionally, its performance was challenged by applying it to a lung radiotherapy treatment very different from the one used for training. The model agreed well with measurements and simulated dose values. A graphical user interface was developed as a first step to making this work more approachable to a daily clinical application.

## 1 Introduction

Radiation therapy (RT) is an effective treatment for cancer. Considering that cancer survival rates have been growing (1) and nearly two-thirds of those survivors are exposed to clinical radiation during its treatment (2), the study of long-term radiation effects, especially secondary cancer induction, has become increasingly important. As many secondary cancers may appear far from the target volumes, the dose received by out-of-field (or peripheral) organs should always be considered for the theoretical secondary cancer risk assessment (3–6).

Unfortunately, up to now, commercial treatment planning systems (TPSs) are not designed for the precise calculation of this peripheral dose, and significant deviations, compared to measurements and/or Monte Carlo (MC) simulations, have been previously reported (7–9). There are several published mathematical models for estimating secondary cancer induction probability as a function of the radiation dose (10–12), which should count with an accurate out-of-field (peripheral) dose distribution received by the patient during RT.

Advanced RT techniques like intensity modulated radiotherapy (IMRT) or volumetric modulated arc therapy (VMAT) are highly effective for achieving tumor control and dose reduction in out-of-field volumes near the border of the field due to reduced internal scatter (13). However, these techniques usually need long beam-on times than conformal treatments, which increase machine scatter and leakage and, consequently, distant peripheral doses. How much the increase in machine scatter and leakage outweighs the internal scatter depends on specific IMRT plans (optimization on the number of monitor units (MUs), tumor size, patient size, etc.). Some studies have quantified the global peripheral dose increase as a 1.8 (14)– 1.9 (15) factor. For volumes distant from the border of the field, where the MU-dependent leakage predominates, a factor of 3 with respect to conformal fields has been found (16).

**Figure 1:**
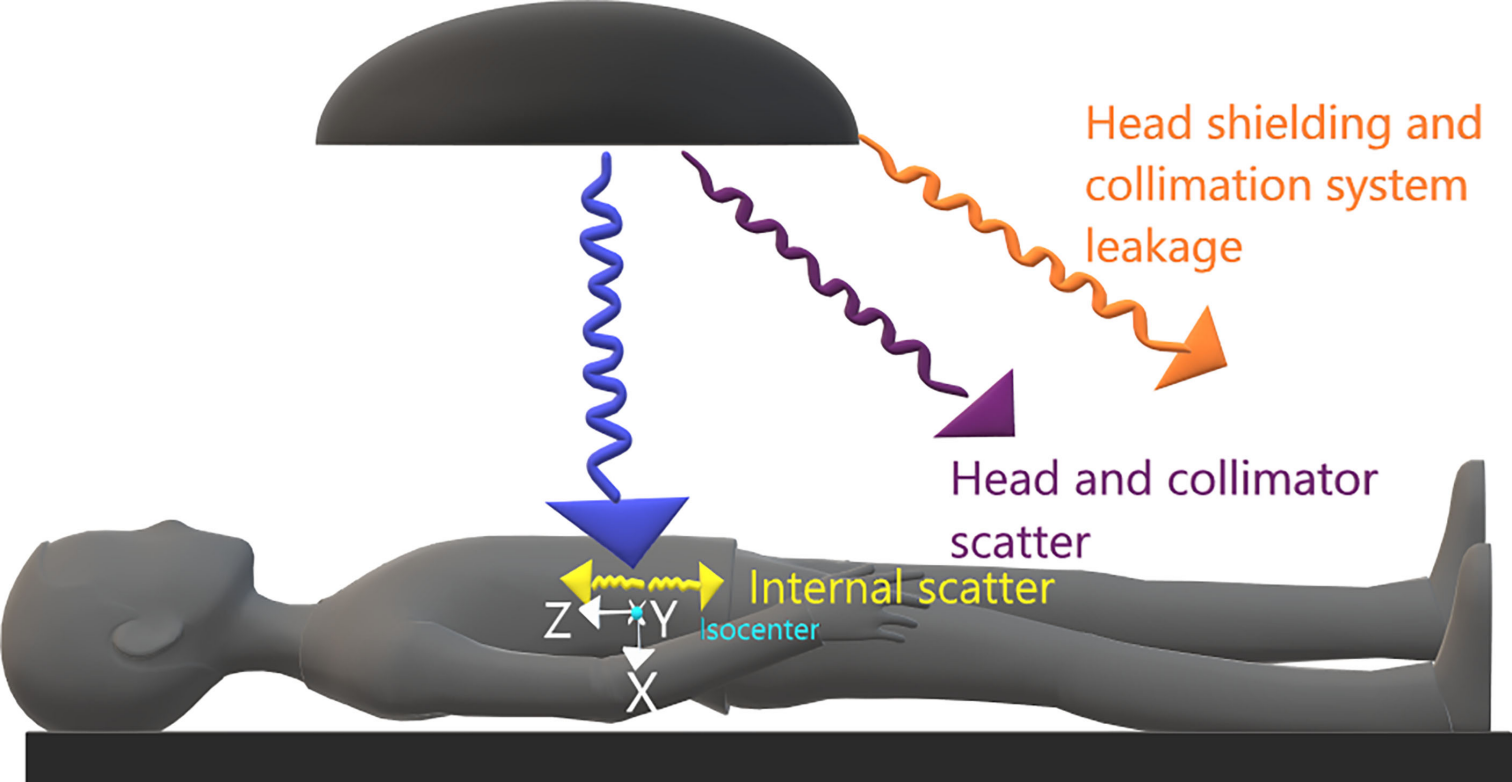
Representation of sources of peripheral dose and coordinate system at the present work.

The peripheral photon dose (PPD) has three sources: i) leakage through the head shielding and the collimation systems, ii) scattering from the head and secondary collimators, and iii) scattering inside the patient (17) (see **Figure 1**). The scattering in the patient is the dominant source of the peripheral dose in regions close to the irradiated volume. However, its relative contribution to the total PPD rapidly decreases for further distances from the treatment edge (considered as the 50% isodose), leaving collimator scattering and leakage as the predominant dose sources in those regions. At considerable distances, leakage is the only relevant dose source (14, 18).

The software Peridose (19) was probably the first attempt for scattered dose calculation outside the primary beam for individual treatments. However, it was only designed to be used for rectangular fields. Then, a simple and flexible analytical model for PPD estimation, also implemented into a computer program termed PERIPHOCAL, correctly predicted the peripheral dose inside a humanoid phantom irradiated with IMRT and VMAT techniques (13, 20). It presents, however, two main limitations: i) the model was trained using only a few measurements points placed inside a humanoid phantom, and ii) it is one dimensional, *i.e.*, it assumed that the organs were described only by the z coordinate of the organ and its length along the craniocaudal direction.

Hauri et al. (21) chose a different approach to model the peripheral dose using complex mathematical functions to represent the physics behind each process and calculate the three peripheral dose components separately. Other recently published models (22, 23) also considered calculating each contribution of the PPD separately. They did calculations in water cylinders with fast computation times but at the price of needing several fitting coefficients. Despite their high accuracy, the main disadvantage of those approaches is their complexity, which makes the clinical application very cumbersome.

In this work, a new analytical model to assess PPD associated with RT is proposed. The model has been trained and validated. It needs only two treatment-specific input variables plus information about the linac-specific leakage when available. As the absorbed dose is given in mGy/MU, the total number of MUs used for the whole treatment will be required for the estimation of the absolute total peripheral absorbed dose. It makes calculations on the whole-body virtual CT of specific patients, which can be generated using a home-made software developed by the authors (24), available upon request. The model has been coded in a piece of software and interacts with the user through a graphical user interface (GUI), making accurate photon peripheral organ dose estimation applicable to the clinical workflow.

## 2 Methods and materials

### 2.1 The analytical model

We propose the following expression to model the 3D distribution of PPD (in mGy/MU):


(Eq.1) $$PPD\left(x,y,z\right)=\left\{\begin{array}{l} \frac{\varepsilon \left(MU\right)\cdot F\left(f\right)\cdot (A_{1}-A_{2}\left|z\right|)\cdot e^{-A_{3}*\sqrt{\;x^{2}+y^{2}+z^{2}}}}{x^{2}+y^{2}+z^{2}}+\left(L_{u}-L_{r}\right);\forall (x,y,z)/\sqrt{\;x^{2}+y^{2}+z^{2}}\leq 40cm\;\;\\ \;\;\;\;\;\;\;\;\;\;\;\;\;\;\;\;\;\;\;\;\;\;\;\;\;\;\;\;\;\;\;\;\;\;\;\;\;\;\;\;\;\;\;\;\;\;\;\;\;\;\;\;\;\;\;\;\;\;\;\;\;\;\;\;\;\;\;L_{u};\;\;\;\;\;\;\;\;\;\;\;\;\;\;\;\;\;\;\;\;\;\;\;\;\;\;\;\;\;\;\;\;\;\;\;\;\;\;\;\;\;\;\;\;\;\;\;\;\;\forall \left(x,y,z\right)/\sqrt{\;x^{2}+y^{2}+z^{2}}>40\;cm \end{array}\right\}$$

where the coordinates *x*, *y*, and *z*  (cm) indicate the position of each calculation point in a coordinate system with the origin at the treatment isocenter (whichever it is). *x, y*, and *z* go in the anterior–posterior, left–right, and caudal–cranial directions, respectively (see **Figure 1**). The model is intended to be used for dose estimation outside the 5% isodose surface from where TPSs are not accurate enough (3, 7). In agreement with other published works (14, 21, 25), our model assumes that for distances to the isocenter larger than 40 cm, the main contribution to PPD comes from the leakage (*L*_*u*_ ), which is considered constant for the purposes of this work.

Equation 1 has some similarities with the model previously proposed by Sánchez-Nieto et al. (13). As in that work, the following correction factors considered here are

● *F*(*f*): It corrects the field size when it is different from the one used in the reference treatment (see *Reference treatment on the ICRP 110 male phantom*) .  This correction is essential as the scattered radiation is field size dependent (25–27). In this work *F*(*f*)=*F*_*U*_(*f*)/*F*_*R*_ , where *F_U_* and *F_R_* are the areas representing the field sizes used in the user and reference treatment plan, respectively. For field size calculation, we propose to take the average of the areas inside the 50% isodoses at the coronal and sagittal planes of the 3D dose distribution at the isocenter level. The estimated value for *F_R_* was 149.2 cm^2^.

● *ϵ*(*MU*): It corrects the number of monitor units (MUs) when they differ from the reference treatment plan (see *Reference treatment on the ICRP 110 male phantom*). This correction accounts that the PPD depends on the number of MUs corresponding to each treatment. In this work, $\epsilon \left(MU\right)=\frac{E_{U}\left(MU\right)}{E_{R}}$ , where *E_U_* represents the efficiency of the user treatment (in terms of the prescribed dose at the isocenter per MU) and, *E_R_* is the treatment efficiency of the reference treatment plan. *E_R_* was calculated, for the calibration conditions of the linac for which the reference treatment was created (1cGy/MU at Source to Surface Distance (SSD), at *d_max_*), as the MU that delivers 2 Gy to the isocenter of the ICRP 110 phantom as if it was made of water ( $\begin{array}{ccc} E_{R}= & 2\cdot \frac{10^{3}Gy}{278\;MU}= & 7.2\frac{mGy}{MU} \end{array}$ )

● *L_u_*: It corrects the leakage value whenever it is different from the one used in the reference treatment (*L*_*r*_ ). This quantity should be measured (in mGy/MU) for every accelerator, but if this parameter is not available, we recommend using the value in this work as an approximation (see *Results*).

The values of the fitted coefficients  *A*_1_(*mGy* *cm* ^2^ *UM* ^−1^) ,  *A*_2_(*mGy* *cm* *UM* ^−1^), and  *A*_3_(*cm* ^−1^)  were obtained by fitting the model to the 3D PPD distribution simulated with MC for the reference treatment plan (see *Reference treatment on the ICRP 110 male phantom* for more details).

In summary, to use this model, the user requires for each calculation point (coordinates in *cm* ), *E*_*U*_(*MU*) in $\frac{mGy}{MU}$ , *F_U_* (*f)* in *cm^2^*, and *L_u_* (when available) in mGy/MU. If the absolute absorbed dose is needed, the total MU will be additionally required.

### 2.2 Reference treatment on the ICRP 110 male phantom

The reference treatment was an equally spaced eight-field isocentric plan centered at the mid-abdomen of the adult reference computational phantom ICRP 110 (28), with 10×10*cm^2^* open fields. The whole-body dose distribution was generated by an MC simulation (BEAMnrc code) of an Elekta Axesse with the Agility collimation system, up to 40 cm from the isocenter. The technical details of the MC simulation can be found in Sánchez-Nieto *et al.* (7). The MC simulation of the ICRP 110 considers the electronic density of each voxel. The uncertainty of the MC dose to points is given directly by the BEAMnrc code within the dose output file (“*.3ddose”) as a relative error value array in row 6 of the file.

### 2.3 Model calibration

Parameters *A*_1_, *A*_2_, and *A*_3_ were obtained by fitting Equation 1 to the 3D MC dose distribution corresponding to the reference treatment (*ϵ* =1 *and F* =1) using the *fminsearch* function in MATLAB^®^ (version R2021a). No information about the electronic density is considered by Equation 1 but the spatial position of the voxels. Only phantom voxels outside the 5% isodose surface were considered for the fitting, as the TPSs accurately estimate the dose distribution inside (7). Voxels representing the body contour were also excluded for the parameterization due to possible electron contamination, which is not considered by this model. As the geometry of the MC simulation did not include the gantry’s shielding, MC data were only used up to 40 cm from the isocenter, and, farther than this point, our measurement of leakage was used instead ( $L_{r}=L_{u}=0.001\frac{mGy}{MU}$ ). The fitting process gave the values of the constant coefficients. *A*_1_(*mGy* *cm* ^2^ *UM* ^−1^), *A*_2_(*mGy* *cm* *UM* ^−1^), and *A*_3_(*cm* ^−1^).

### 2.4 Dose to organs

The model in Equation 1 depends on the three Cartesian coordinates; therefore, when the calculation is made on a whole-body CT, the model generates a 3D out-of-field dose cube from which the dose–volume histogram (DVH) of the contoured organs can also be extracted.

### 2.5 Experimental validation

#### 2.5.1 Validation using TLD-100 in an anthropomorphic phantom

We first tested the model by applying it to a case of the pelvic irradiation of an anthropomorphic phantom and comparing the results with TLD-100 measurements. A 6 MV VMAT treatment for prostate cancer was planned (MONACO) and delivered to the male 701-D ATOM phantom (CIRS^®^) with an Elekta Synergy linac (different from the one used as reference). The phantom, which only consists of the head and torso, held 271 TLD-100 chips distributed in 20 predefined internal organs. The Thermoluminiscent Dosimeter (TLDs) had been previously calibrated using one X-ray equipment with beam quality corresponding to an Half Value Layer (HVL) = 6.141 mm Al. Energy corrections according to the mean energy at each point (7) were applied following Duggan’s model (29). The ATOM phantom was previously scanned for planning. Then, on the planning station, the prostate gland and rectum outlines were drawn following the contours of a real plan of another prostate cancer patient with similar physical characteristics. Finally, a VMAT plan was created to deliver one fraction of 1.8 Gy at the isocenter, corresponding to a total of 498 MU (i.e., *E_U_* = 3.6 mGy/MU). *F_U_* was equal to 53.2 cm^2^ in this case (calculated as the average of the areas inside the 50% isodoses at the coronal and sagittal planes of the 3D dose distribution at the level of the isocenter). The absorbed dose to each point measured by the TLDs was compared to our model predictions using the following values of the model variables: $\epsilon =\frac{1.8*10^{3}/498}{2.10^{3}/278}=\frac{3.6}{7.2}=0.48$ and $F=\frac{53.2}{149.2}=0.35$ and *L*_*u*_=0.0032 *mGy*/*MU* (measured with TLD-100 at 40 cm from the isocenter).

Uncertainty in the dose estimated by TLD measurements was calculated from the propagation of the variables´ uncertainty involved in dose calculation (i.e., experimental TLD calibration, individual sensitivity, and energy correction factors).

#### 2.5.2 Testing the model using a Monte Carlo simulation of a lung treatment on the ICRP phantom

The performance of the model was challenged by applying it to a case very different from the reference treatment: a three-field equally weighted lung irradiation plan (5×5  cm^2^ open fields) with one AP (60°) and two posterior oblique (220° and 240°) fields. The plan was simulated with MC on the ICRP110 reference phantom (7). The treatment isocenter was located at the upper-right lung lobe receiving 2 Gy per fraction. The whole-body dose distribution was obtained and compared with the estimations of the model presented in this work. The *ϵ* and *F* values used for the model estimations were $\epsilon =\frac{2*10^{3}/266}{2.10^{3}/278}$ =1.04, $F=\frac{53.41}{149.2}=0.36$ and $L_{u}=L_{r}=0.001\frac{mGy}{MU}$ .

The absorbed dose to organs calculated with the proposed model, the MC simulation, and the software PERIPHOCAL (13) were also compared, using the same leakage and field size for the modeling cases. The same organs considered by PERIPHOCAL were selected for comparison. The PERIPHOCAL model calculates dose uncertainty ranges (95% confidence interval) using the expression 7 of the publication (13). The contours for those organs were taken from the ICRP 110 phantom.

## 3 Results

### 3.1 Reference treatment and model calibration

**Figure 2:**
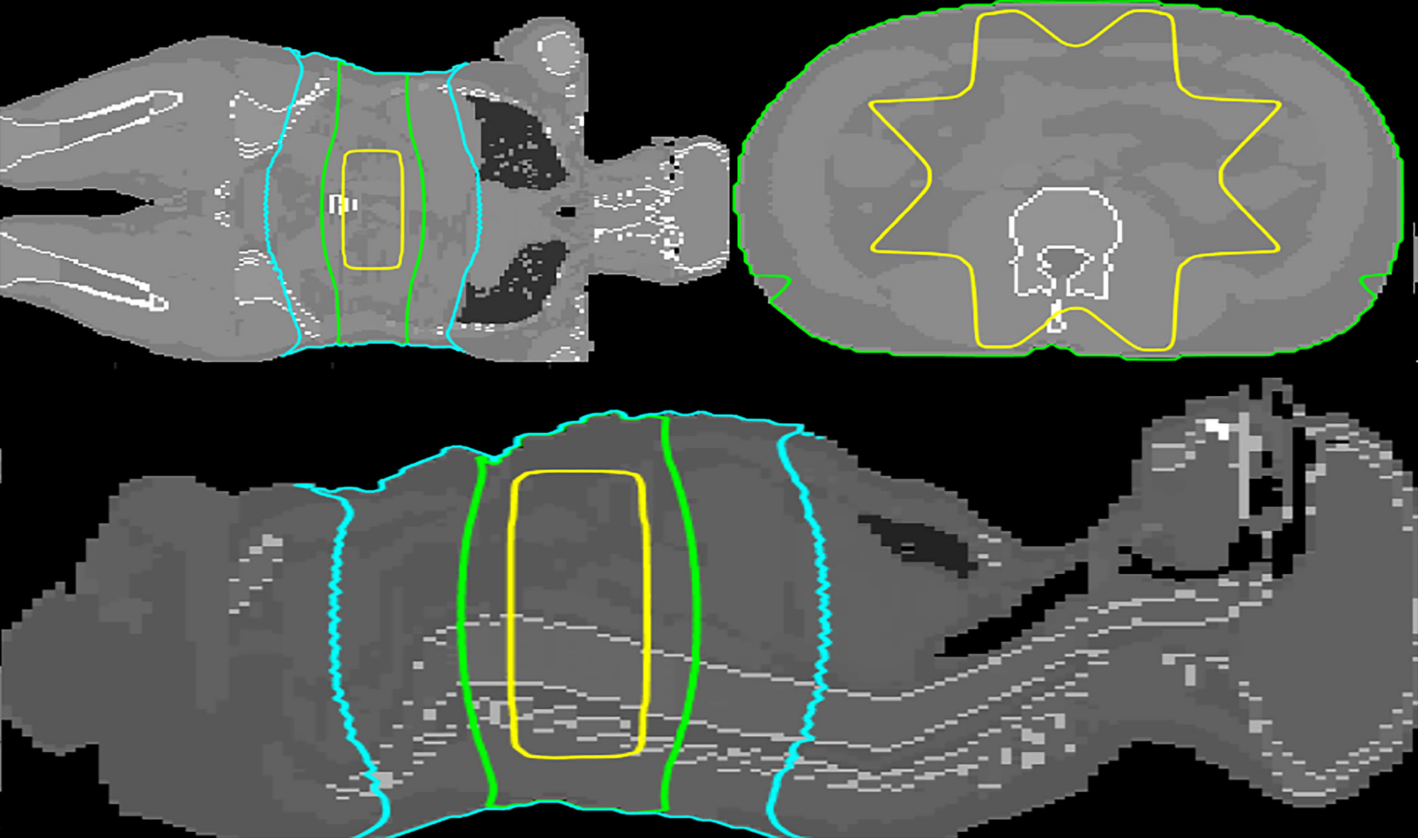
Transversal, coronal and sagittal views at the level of the isocenter (mid-abdomen). The 50%, 5%, and 1% isodoses are depicted in yellow, green, and cyan, respectively. The dose distribution was calculated by MC simulation.

Representative isodoses of the reference treatment plan on the ICRP 110 phantom are shown in **Figure 2**.

The fitted constant coefficients are (*Model calibration*) $\;A_{1}=37.890\;\pm \;1.415\;(\frac{mGy\;cm^{2}}{MU})$ , $A_{2}=0.679\pm 0.074\;(\frac{mGy\;cm}{MU})$ , *A*_3_ = 0.007 ± 0.004 (*cm* ^−1^).

The final (calibrated) version of the model can therefore be written (for points outside the 5% isodose) as in Eq (2).


(Eq.2) $$PPD\left(x,y,z\right)=\;\{\begin{array}{l} \frac{\frac{E_{U}(MU)}{7.2\frac{mGy}{MU}}\;\cdot \;\frac{F_{U}\mathrm{(f)}}{149.2\;\text{cm}2}\;\cdot \left(37.890\frac{mGy\;cm^{2}}{MU}\;-\;0.679\;\frac{mGy\;cm}{MU}*\left|z\right|\right)\cdot e^{-0.007*\sqrt{\;x^{2}+y^{2}+z^{2}}}}{x^{2}+y^{2}+z^{2}}+\left(L_{u}-0.001\frac{mGy}{MU}\right);\forall \left(x,y,z\right)/\sqrt{\;x^{2}+y^{2}+z^{2}}\leq 40cm\;\\ \;\;L_{u};\;\;\;\forall \left(x,y,z\right)/\sqrt{\;x^{2}+y^{2}+z^{2}}>40\;cm \end{array}\}$$

The uncertainty of the model was calculated considering the absolute percentage differences between the doses given by the model and the ones given by MC, relative to MC. In total, 95% of all points (x, y, and z) presented an absolute percentage difference<23.2% (the average percentual difference was 7.84%).

**Figure 3:**
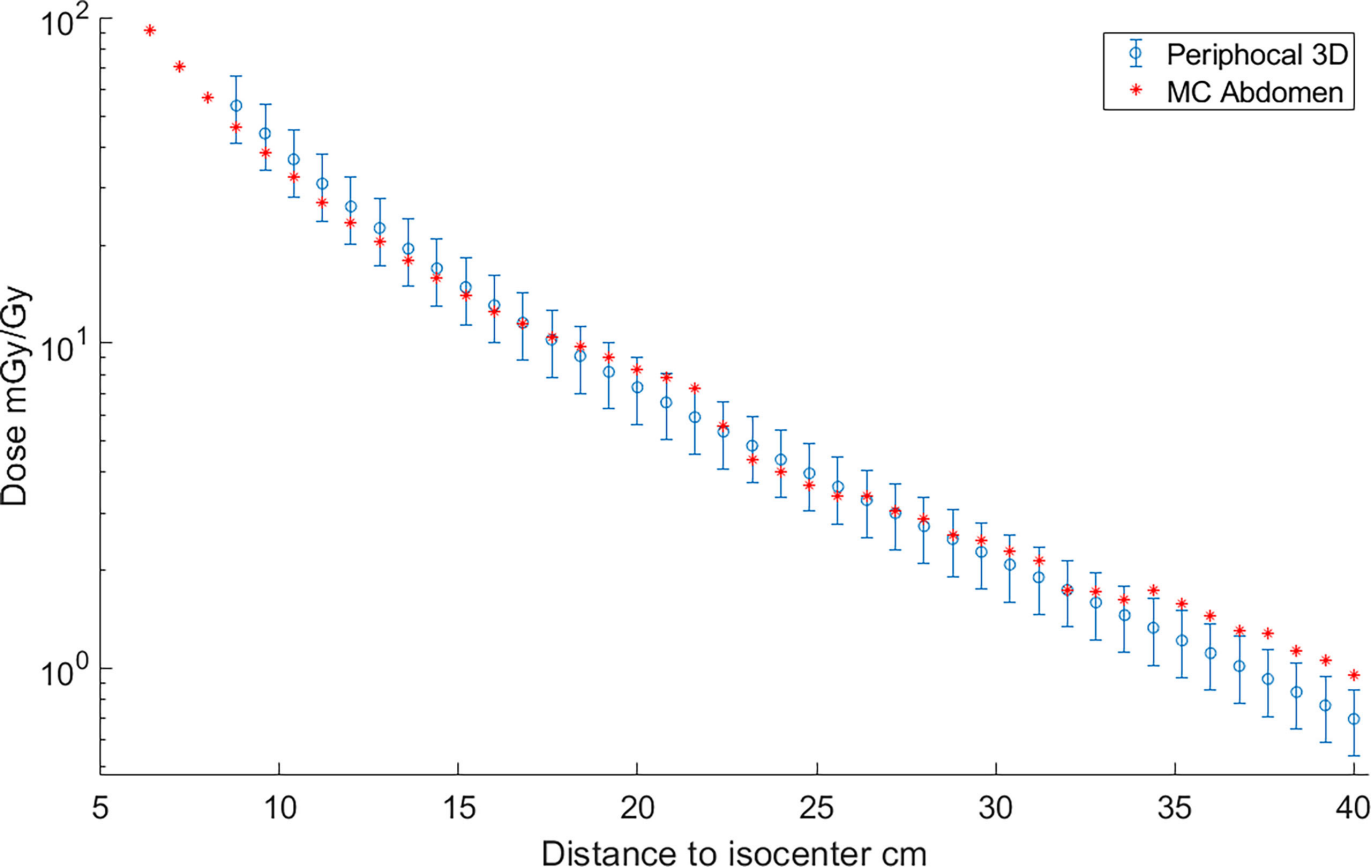
Absorbed dose, expressed as peripheral dose (mGy) given by both Periphocal 3D and the MC simulation for the reference plan on the ICRP 110 phantom, relative to the isocentric dose (Gy) vs. distance to isocenter. Displayed symbols correspond to points along the craniocaudal axis (towards the phantom’s head) at the isocenter depth. The uncertainty associated with Periphocal 3D is ± 23.2%. The uncertainties of the MC dose values are within the size of the symbols. Even though the model was parameterized using the dose distribution calculated by MC, which considers the electronic density of each voxel, and the analytical model assumes a uniform electronic density, there is an agreement for most of the points.

Hereafter, the model in Equation 2 will be named Periphocal 3D. **Figure 3** depicts the peripheral dose to points relative to the isocentric dose, estimated by Periphocal 3D and MC used for calibration. Note that this model does not use any electronic density information.

### 3.2 Model validation and testing

#### 3.2.1 Validations using TLD-100 inside the ATOM phantom

**Figure 4:**
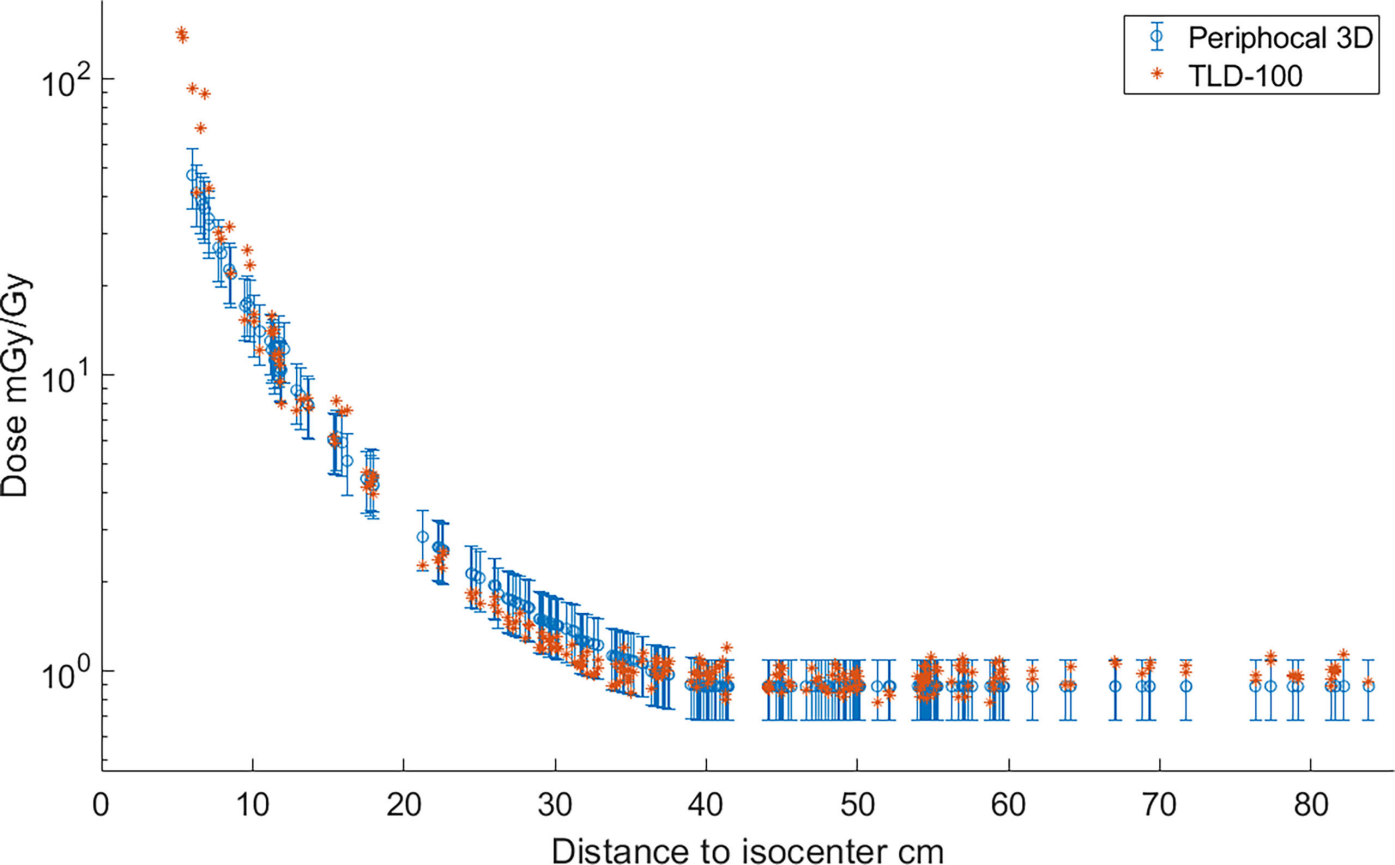
Absorbed dose, expressed as peripheral dose (mGy), calculated by Periphocal 3D and measured with TLD-100, relative to the isocentric dose (Gy) for a VMAT irradiation of the prostate. As TLDs positions are scattered inside the ATOM phantom, the dose values were plotted versus Euclidean distance to the isocenter. TLDs uncertainties are within the size of the symbol.

Measurements obtained with the TLDs and the dose estimated by Periphocal 3D for the same positions are depicted in **Figure 4**. The average absolute difference between Periphocal 3D dose estimations and the TLD dose measurements, relative to the latter, is 16.8%, with a maximum difference on one point of 15.8 mGy/Gy (31.4 mGy/Gy predicted by the model, 47.2 mGy/Gy measured by TLD). The model performance is in the low extreme of mean differences of 11%–44% mentioned in Mazonakis and Damilakis (3).

#### 3.2.2 Testing in a more complex scenario

**Figure 5:**
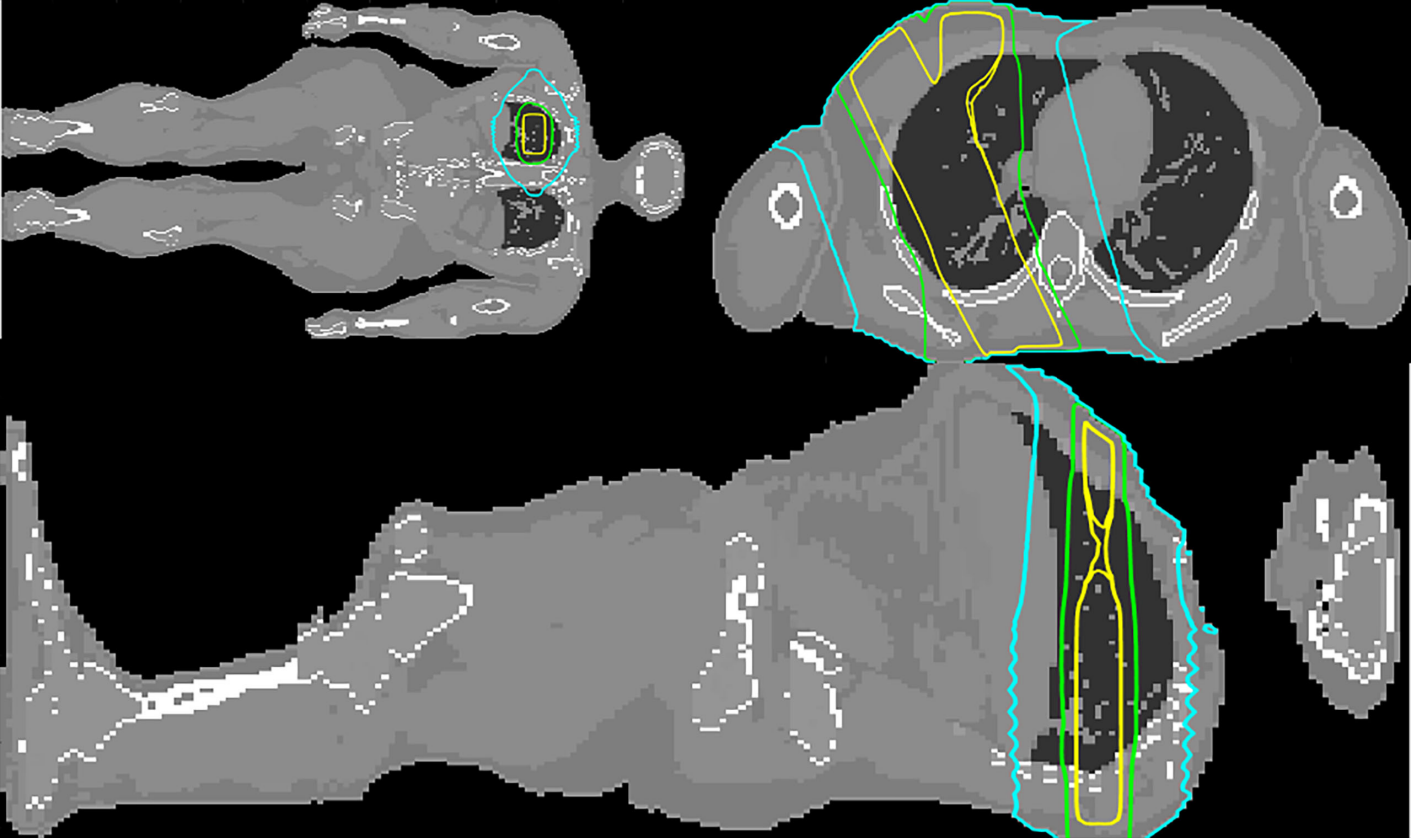
Coronal, transversal and sagittal views at the level of the isocenter of the MC simulated upper right lung irradiation with three square beams of . The 50%, 5%, and 1% isodoses are depicted in yellow, green, and cyan, respectively.

**Figure 6:**
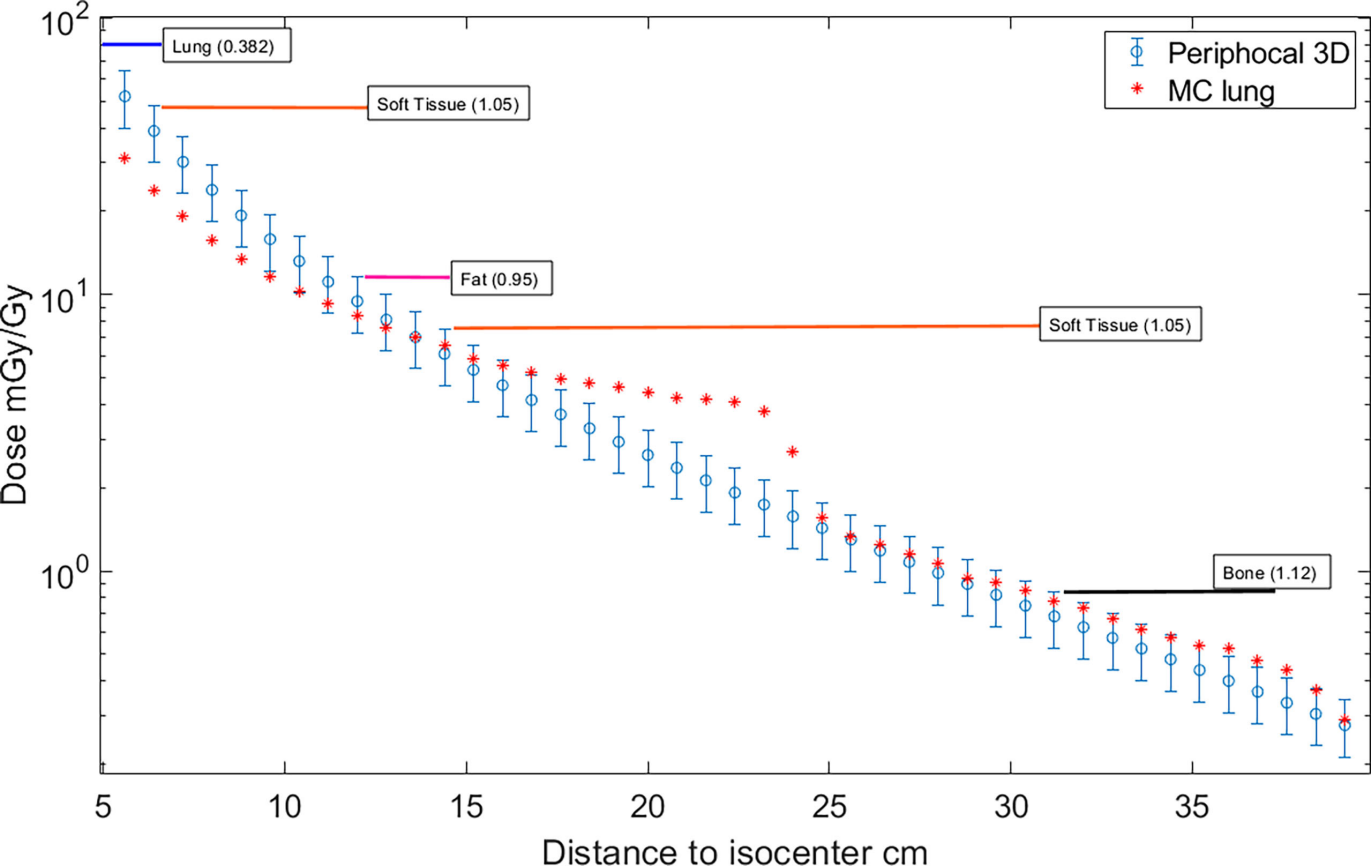
Absorbed dose, expressed as peripheral dose (mGy), calculated by Periphocal 3D and MC for the lung case, relative to the isocentric dose (Gy). Displayed symbols correspond to points along the craniocaudal axis (towards the phantom’s feet) at the isocenter depth. The uncertainties of the MC dose values are within the size of the symbols. It has also been displayed the type of tissue together with the corresponding electronic density in which calculations were performed (e.g., from 7 cm to 12 cm far from the isocenter is soft tissue).

Representative isodoses calculated with MC on the ICRP 110 phantom, corresponding to the lung plan described in *Testing the model using a Monte Carlo simulation of a lung treatment on the ICRP phantom*, are shown in **Figure 5**. **Figure 6** depicts the peripheral dose, relative to the isocentric dose, calculated by Periphocal 3D and MC for the same case.

The average of absolute differences relative to MC is 44.0%, with a maximum difference of 34.9 mGy/Gy on a point (14.1 mGy/Gy predicted by the model and 49.0 mGy/Gy simulated by MC).

**Figure 7:**
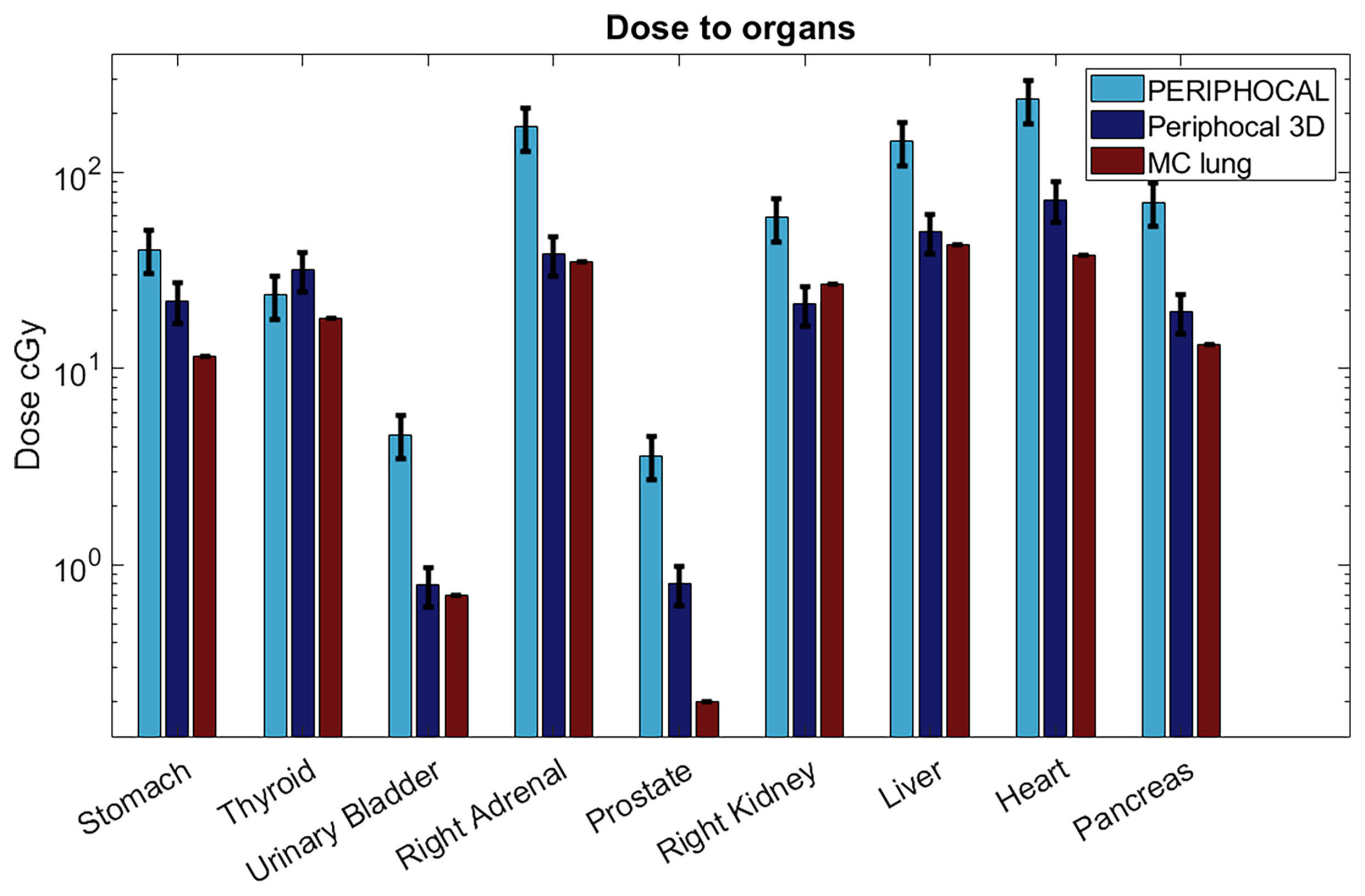
Absorbed dose to organs according to Periphocal 3D, PERIPHOCAL, and the MC simulation of the lung plan for the 60 Gy delivered to the isocenter. Error bars are presented as the uncertainty in each model (25% for PERIPHOCAL and 23.2% for Periphocal 3D).

The comparison of absorbed dose to a set of organs, given by PERIPHOCAL (13), Periphocal 3D, and the MC simulation, is shown in **Figure 7**.

## 4 Discussion

Periphocal 3D gives the PPD in 3D as a function of the point’s coordinates, and it requires only three input treatment parameters: the field size, total MU, and the MU per Gy to the isocenter (*L*_*u*_ is the fourth parameter, which can be used when available). It has three empirically fitted coefficients *A*_1_ , *A*_2_ , and *A*_3_. Even though those coefficients do not have a direct physical meaning, *A*_3_  may be seen as an ‘effective’ linear attenuation coefficient of photons scattered inside and outside the patient.

### 4.1 Limitations of the model

Although Periphocal 3D represents an improvement compared to its previous version, there are some limitations to take into account. Regarding geometry and X-ray attenuation, it is worth noting that Periphocal 3D was calibrated with a nearly symmetrical eight-field treatment (see **Figure 2**) and Equation 1 has spherical symmetry. Thus, the model should become less accurate when non-symmetrical isodoses, usually associated with plans with fewer beams, as in **Figure 5** (lung case), are generated. Luckily, VMAT and IMRT treatments usually consider several beam entries around the isocenter. Another possible issue is the attenuation of X-rays inside the patients when significant tissue heterogeneities are present. Since Periphocal 3D was calibrated in the abdomen, which is mainly composed of soft tissue, its application to the treatments of tumors in tissues with different X-ray attenuation characteristics will necessarily imply worse accuracy. This can be observed in **Figure 6**, where calculations within the soft tissue after the lung shows an overestimation of the dose because it does not take into consideration the smaller backscatter contribution from the lung tissue. Additionally, in bone, as Periphocal 3D assumes that everything is water, the calculated absorbed dose underestimates the MC calculation.

It is worth noting that the large discrepancy between MC and Periphocal 3D calculations in the lung plan is mainly associated with the use of small fields (5 × 5 cm^2^) for which a sharp drop at 21 or 22 cm approximately from the isocenter occurs in correspondence with the edges of the primary collimator that provides additional shielding. The same phenomenon has been described elsewhere (30, 31). This drop is much softer for larger beams, as seen in **Figure 3** for the 10 × 10 cm^2^, in agreement with Kaderka et al. and Jagetic and Newhauser (30, 31). That is, the full MC simulation recreates a profile shape that the Periphocal 3D does not (see *Comparison with another analytical model* to verify how a more complex physics-based model also fails in fitting the decrease due to the additional shielding of the primary collimator).

The *A*_1_, *A*_2_, and *A*_3_ coefficients were established by fitting the model to the dose distribution generated by the MC simulation of an Elekta Linac. Stoval et al. (18) showed in Appendix A how peripheral dose profiles depend on the design and construction of the machine head and collimators. However, our measurements—figure 1.a in Sánchez-Nieto et al. (13)—of peripheral doses for the same treatment delivered on the same phantom using different combinations of energy and linacs allowed us to conclude that the observed variability was within the model´s uncertainty. Those who can generate a 3D dose distribution of their specific linac from a full MC model or thorough experimental measurements can obtain their specific *A*_1_, *A*_2_, and *A*_3_ coefficients.

Periphocal 3D does not work for skin dose calculations. A peripheral skin dose can be separately estimated based on other previously published works (32).

### 4.2 Dose to organs

The availability of 3D dose distribution allows for the calculations of DVHs for peripheral organs (input for some models of secondary cancer risk), dose profiles along any axis or 2D dose distributions on any plane. The 3D dose distribution may also be helpful, for example, when the patient has a pacemaker, an intern defibrillator at positions where TPSs are not accurate, or for any of the harmful effects listed by Mazonakis and Damilakis (3).

**Figure 7** shows the dose to organs for a lung treatment in comparison to PERIPHOCAL and MC. This is a case for which Periphocal 3D presents some limitations, as discussed in the previous section. Even so, the model offers an improvement compared to PERIPHOCAL for all organs but the thyroid. Both PERIPHOCAL and Periphocal 3D overestimate the dose, which can be explained by the highly non-symmetrical geometry of the dose distribution in this area. For the prostate, despite Periphocal 3D performing much better than PERIPHOCAL, a significant difference compared with MC is still present. As we already mentioned, the geometry of our MC did not include the gantry’s shielding and, therefore, might not correctly account for leakage. Thus, the dose for the prostate (farther away than 40 cm from the isocenter) given by MC may be underestimated. A detailed study of the effect of linac’s shielding on the leakage is being conducted. For the urinary bladder, close to the prostate but closer to the isocenter than 40 cm, Periphocal 3D and MC agree.

This model can be used retrospectively and prospectively (for example, using the virtual whole-body CT generated by our home-made software (24) to calculate in a systematic way dose to peripheral organs and, together with clinical follow-ups, detecting possible secondary cancers, creating a database for a more accurate parameterization of secondary cancer models (3–6).

Sánchez et al. (13) showed that PPD does not significantly change with energy (differences within the model´s uncertainty), and thus, Periphocal 3D can be used even above 10 MV. However, neutron contamination might become relevant above this threshold. For those dealing with energies >10 MV, the total peripheral dose to organs should include the peripheral neutron dose to organs. The model published by Irazola et al. (33) can be used to estimate the neutron dose to out-of-field organs.

### 4.3 Comparison with another analytical model

Schneider et al. (22) developed a nice physics-based analytical model of the total absorbed dose for the primary, scattered, and leakage radiation of square fields of 6 MV at any arbitrary point in a phantom. That work is one of the latest models published but tested only for square fields. They mention the validation of the model for arbitrary MLC aperture to determine the model applicability to IMRT treatments as a future step. Thus, to our knowledge, no other analytical model has been developed and tested for intensity-modulated treatments with which we can compare.

**Figure 8:**
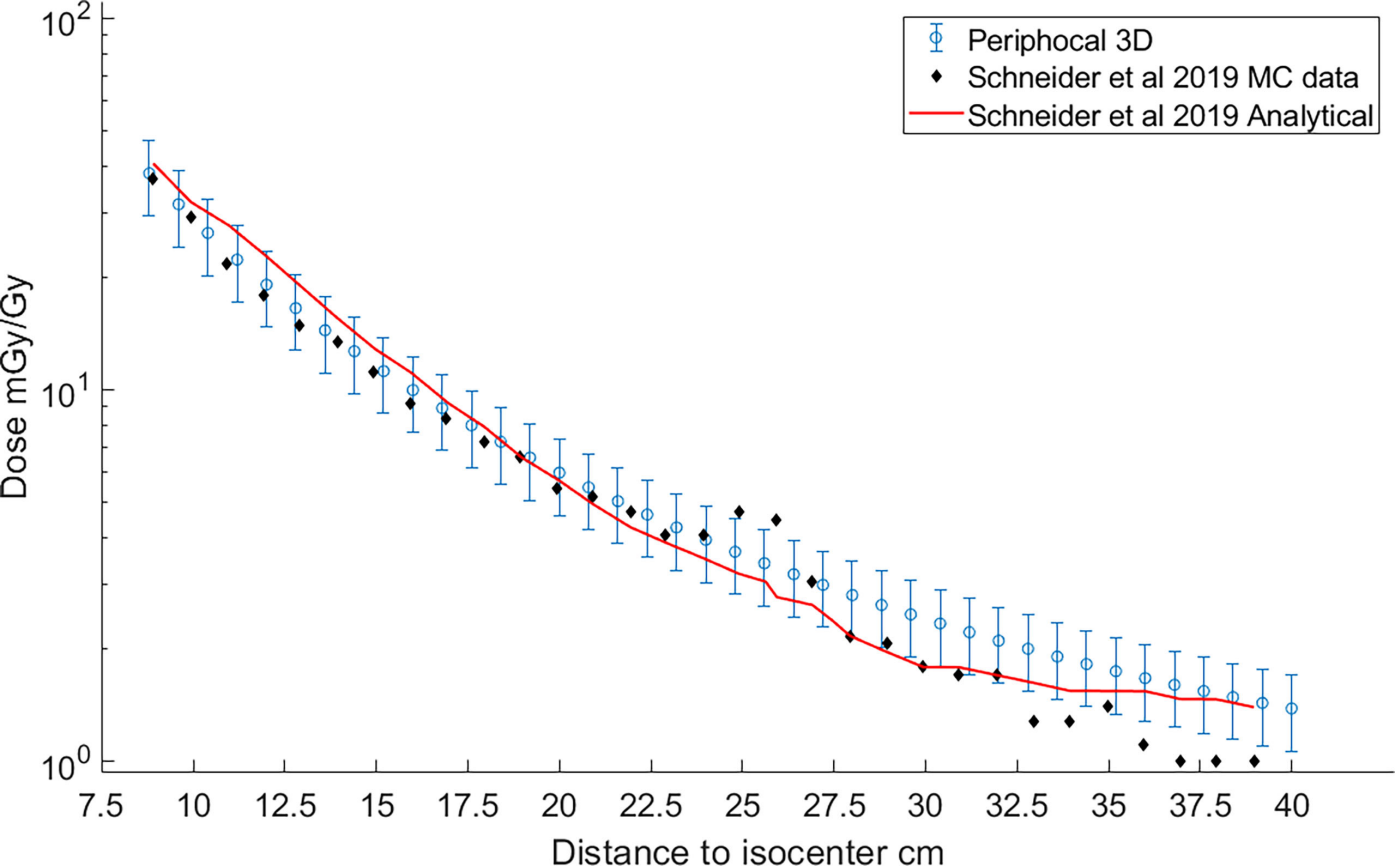
Comparison of the peripheral dose calculated with Periphocal 3D (light-blue open symbols with uncertainty bars) and Schneider et al. (22) model (solid line). Schneider model´s data were recreated from figure 14.a of the publication (22). Additionally, the MC data used to fit the 30 parameters of the peripheral dose of Schneider´s model—table 4 in Schneider et al. (22)—are also included as black diamonds.

**Figure 8** depicts the comparison between the dose as calculated by our model and Schneider´s model for irradiation with a 10×10*cm* ^2^ field using a 6 MV beam. The parameter field size *F_U_* (*f)* at the isocenter was estimated from the width of the profile at 50% of the isocenter dose as *F*_*U*_(10)=103.63 *cm* ^2^ . It was also assumed that ϵ*_U_* = ϵ*_R_*

Both models agree within our model´s uncertainties. Curiously enough, both models fail to reproduce the additional shielding of the primary collimator as predicted by Schneider´s MC model. Note that this effect is sharper for smaller fields, as in the case of **Figure 6**, corresponding to a treatment plan of 5×5 cm^2^.

### 4.4 Whole-body CT

In this work, the ICRP 110 phantom and the CT of the ATOM phantom were used for the 3D calculations. However, for personalized clinical applications, a whole-body CT of each patient is needed. We have solved this problem without actually irradiating the patient (which would be unacceptable due to the unjustified additional dose), following a methodology presented in the companion article. This methodology uses the always-available planning CT to generate an approximate patient-specific whole-body CT based on a rigid 3D image registration algorithm. The input for calculating the 3D dose distribution is the whole-body CT ignoring the differences in electronic density between the voxels but with all organs’ contours considered in the ICRP110.

### 4.5 Graphical user interface

**Figure 9:**
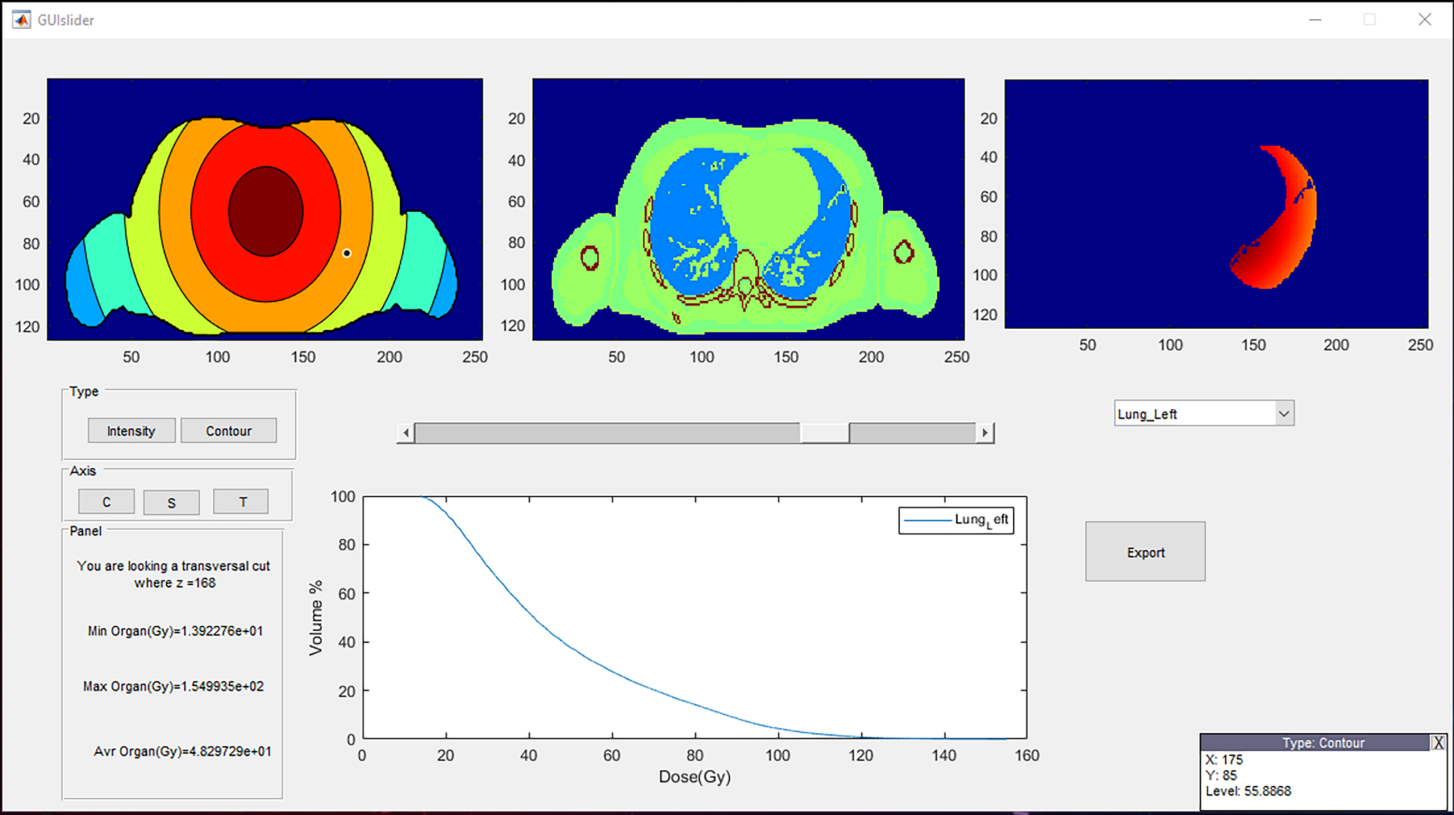
A representative visualization of the Periphocal 3D’s GUI. The three upper boxes can be displayed as transversal (T), coronal (C), or sagittal (S) views. The upper-left box represents the peripheral dose normalized to the maximum (which is always displayed in red) (i.e., the red color might represent a different level of dose at each different slice), and the central box is the anatomical information. The upper-right box represents the dose distribution of the chosen organ (lung in this example) from the list of all contoured organs. Its corresponding cumulative DVH is displayed in the plot below. The DVH can be exported as an ASCII file. The lower-left box informs the value of the *z* coordinate, together with the maximum, minimum, and average dose of the chosen organ. The user can finally move around the upper-left and central boxes so that the coordinates and dose level (in mGy/Gy) of the cursor are displayed in the lower-right box. The GUI can be shared with those who request it.

A GUI was created in MATLAB^®^ (version R2021a) to ease the use of Periphocal 3D (**Figure 9**). The output of our whole-body CT software is a MATLAB array. Periphocal 3D’s GUI takes less than 10 s to calculate the whole-body dose distribution after loading those MATLAB arrays. However, Periphocal 3D’s GUI also accepts CT DICOM files. Additionally, for all organs segmented in the input CT, a DVH is created. **Figure 8** depicts an example of the mentioned GUI.

## 5 Conclusions

A simple 3D analytical model was created for photon peripheral dose estimation outside the 5% isodose for isocentric coplanar treatments with any field sizes (with or without intensity modulation) and applicable to all linacs manufacturers. The model was successfully tested with experimental dose measurements on an anthropomorphic phantom irradiated with a VMAT treatment plan and compared with one physics-based analytical model. It only uses three (or four) input parameters to characterize each radiotherapy treatment. As a first step to making this work more approachable to a daily clinical application, a graphical interface was developed, making the calculation of DVHs in peripheral organs and the 3D visualization of the corresponding dose distributions possible.

## Data availability statement

The standalone software Periphocal 3D and the ICRP 110 adult reference phantom, with the format required by Periphocal 3D, are available upon request to bsanchezn@uc.cl.

## Author contributions

BS-N designed the project, got the funding, co-supervised the work of IL-M, and wrote the paper. IL-M worked in the development of the model under the guidance of BSN and IE. JR-M brought out the Monte Carlo Simulations and help IL-M to develop the model. IE co-supervised the work of IL-M and collaborated with the writing of the manuscript. All authors contributed to the article and approved the submitted version.

## Funding

This work was funded by ANID (FONDECYT N1181133).

## Acknowledgments

We would like to acknowledge Jessica Hernández and Gabriel Zelada (Clínica Alemana-Universidad del Desarrollo, Santiago, Chile) for the data set of TLD-100 measurements.

## Conflict of interest

The authors declare that the research was conducted in the absence of any commercial or financial relationships that could be construed as a potential conflict of interest.

## Publisher’s note

All claims expressed in this article are solely those of the authors and do not necessarily represent those of their affiliated organizations, or those of the publisher, the editors and the reviewers. Any product that may be evaluated in this article, or claim that may be made by its manufacturer, is not guaranteed or endorsed by the publisher.
